# PS341 inhibits hepatocellular and colorectal cancer cells through the FOXO3/CTNNB1 signaling pathway

**DOI:** 10.1038/srep22090

**Published:** 2016-02-26

**Authors:** Zhao Yang, Shengwu Liu, Mingao Zhu, Hong Zhang, Ji Wang, Qian Xu, Kaisu Lin, Xiumin Zhou, Min Tao, Chong Li, Hong Zhu

**Affiliations:** 1 Department of Oncology, the First Affiliated Hospital of Soochow University, Suzhou, 215006, China; 2 CAS Key Laboratory of Pathogenic Microbiology and Immunology, Institute of Microbiology, Chinese Academy of Sciences, Beijing 100101, China; 3 Department of Medicine, Harvard Medical School, Boston, MA 02115, USA; 4 Department of Medical Oncology, Dana-Farber Cancer Institute, Boston, MA 02215, USA; 5 Department of Oncology, the Second Affiliated Hospital of Soochow University, Suzhou, 215000, China; 6 Laboratory Animal Center, Institute of Biophysics, Chinese Academy of Sciences, Beijing 100101, China; 7 CAS Key Laboratory of Infection and Immunity, Institute of Biophysics, Chinese Academy of Sciences, Beijing 100101, China

## Abstract

Hepatocellular carcinoma (HCC) and colorectal cancer (CRC) are among the most common cancers across the world. Particularly, a large number of patients with CRC also have liver metastasis. Currently, there are just a few targeted drugs against these two kinds of tumors which can only benefit a very small population of patients. Therefore, the need of more effective therapeutic drugs or strategies for these two types of cancers is urgent. PS341 (Bortezomib) is the first proteasome inhibitor drug which has been approved in clinical treatment for multiple myeloma. Here we demonstrated that PS341 negatively regulated HCC and CRC both *in vitro* and *in vivo*, including the inhibition of cell proliferation, epithelial-mesenchymal transition (EMT), the expression of stemness-related genes, cell migration and invasiveness. Mechanically, PS341 upregulated the expression of FOXO3, which inhibited the transcriptional activation of *CTNNB1*. The downregualtion of CTNNB1 led to apoptosis, cell cycle arrest, and the inhibition of migration, invasion, self-renewal and tumor formation of these two cancer types. In sum, our findings shed light on the PS341 mediated targeted therapy against both HCC and CRC in the future.

Hepatocellular carcinoma (HCC) is the sixth most common cancer worldwide and the most common liver cancer^1^. Due to high prevalence of HBV and HCV infection, alcoholism and obesity, the incidence of HCC is dramatically increasing in the US, Europe and Asia^1^,^2^. Surgery is still the current mainstay of HCC treatment. Unfortunately, there was a dramatic reduction in the mortality only in some well-selected HCC patients. While in other patients, the rates of tumor metastasis and recurrence were still very high and these patients showed a relative short lifespan even after surgery. Moreover, in the aspect of chemical treatment, HCC is non-sensitive to most of anticancer drugs for either genetic or acquired chemoresistance^2^,^3^. Therefore it is of high importance to develop a targeted HCC therapy which can interfere with some specific molecules in tumor cell proliferation and survival. An oral inhibitor for multi kinases, Sorafenib, was approved in USA and Europe to treat advanced HCC patients^4^. However, there is not a satisfactory role that Sorafenib is playing in treatment of other stages of HCC^5^. So it is highly meaningful to develop novel and potent molecular targeted drugs for HCC.

In western countries such as Europe and USA, primary HCC is less common than liver metastases which mostly develop from colorectal cancers (CRCs)^6^.Colorectal cancer is one of the largest reasons for morbidity and mortality caused by cancers^6^,^7^. Liver diseases were found in about 77% of patients affected with metastatic CRCs^6^. There are currently three drugs together with four available drugs for targeted therapy against metastatic colorectal cancer^8^. However, all these drugs can only benefit a small group of patients depending on their genetic signatures and thus many new targeted drugs are in urgent need to be developed in the future^8^.

PS341 is not only the first proteasome inhibitor applied in clinical treatment but also the only molecular targeted drug which has been approved to treat recurrent and refractory multiple myeloma^9^. PS341 can suppress the proteasome-mediated degradation of pro-apoptotic and anti-apoptotic proteins, thus directly inducing cancer cell apoptosis^10^. Moreover, it also downregulates VEGF expression to indirectly inhibit angiogenesis in multiple myeloma^10^,^11^. Based on its inhibitory mechanism PS341 is also suitable for many other solid tumors besides multiple myeloma^9^. Although some studies indicated that PS341 either alone or in combination had unsatisfactory clinical activities in phase II studies in patients with hepatocellular carcinoma or colorectal cancer^12^,^13^. However, there is little research fully clarifying the mechanisms of PS341 in HCC and CRC therapy.

Here in our research, we aimed to explore whether PS341 can efficiently inhibit the proliferation, migration and invasion of HCC and CRC cells and disclose the influence of PS341 on HCC and CRC cells. Our results showed clearly that PS341 could significantly suppress the proliferation, migration, EMT and the expression of stemness-related gene of HCC and CRC cells both *in vitro* and *in vivo* with the upregulation of FOXO3 and downregulation of CTNNB1, which shed some light on the future HCC and CRC treatment based on PS341.

## Results

### PS341 specifically inhibits the growth and promotes the apoptosis of HCC and CRC cells

**Figure 1:**
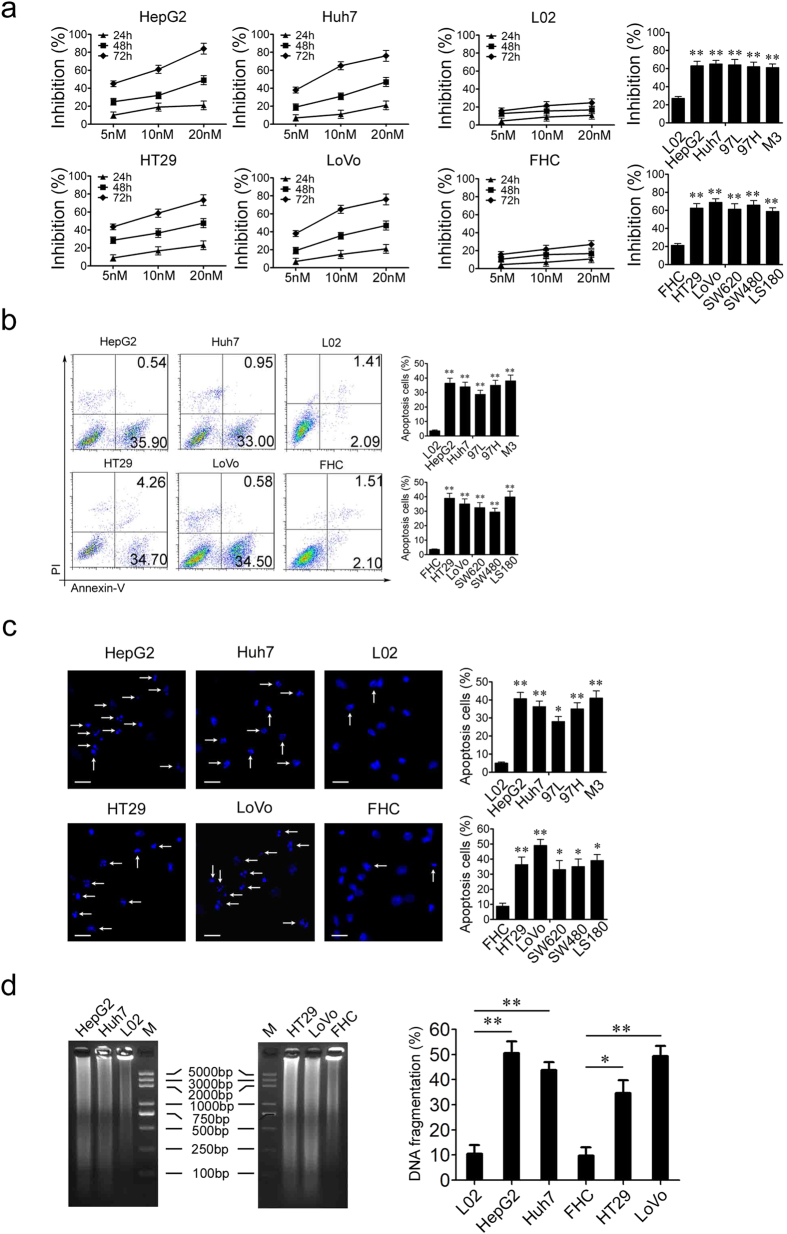
PS341 specifically inhibits the growth and promotes the apoptosis of HCC and CRC cells. (**a**) PS341 inhibits HCC and CRC cell growth in a time and dose dependent manner. 5 nM, 10 nM or 20 nM PS341 were introduced into the medium of L02, HepG2, Huh7, FHC, HT29 and LoVo cells for 24 h, 48 h and 72 h. The inhibition of cell viabilities was measured by the method of CCK8. (**b**) PS341 induces apoptosis of HCC and CRC cells. The apoptosis cells were detected with Annexin V-FITC and PI staining. (**c**) PS341 induces apoptosis identified by Hoechst 33342 staining. The cells were fixed with 4% paraformaldehyde and stained with Hoechst. Scale bar = 25 μm. (**d**) PS341 induces apoptosis identified by DNA ladder assay. The whole assay was repeated three times. Data are presented as mean ± SD. *P < 0.05, **P < 0.01.

In order to identify the potential role of PS341 in HCC and CRC therapy, we first examined the function of PS341 in two HCC cell lines HepG2 and Huh7 and two CRC cell lines HT29 and LoVo, respectively. We cultured each cell line with different concentration of PS341 for 24, 48 or 72 h and then analyzed the cell viability with the method of cell counting kit-8. The growth of both HCC and CRC cells (Fig. 1a, left) was inhibited by PS341 in a dose and time dependent way. In contrast, cell growth remained almost the same for an immortalized normal liver cell line L02 and a normal colon cell line FHC (Fig. 1a, middle). In the following, more HCC and CRC cells were tested with 10 nM PS341 for 72 h. It indicated the cell proliferation of HCC lines 97L, 97H and M3 and CRC cells SW620, SW480 and LS180 were also inhibited remarkably (Fig. 1a, right). These data showed that PS341 specifically suppressed the growth of HCC and CRC cells but not normal cells *in vitro*.

Apoptosis and mitosis are closely linked and balanced in cell cycle. We further investigated the role of PS341 in cell death of HCC and CRC cells. Using FACS assay with Annexin V/PI staining, we found apoptosis could be significantly induced only in HCC and CRC cells but not within normal cell lines L02 and FHC (Fig. 1b) when treated with PS341. What’s more, we also observed dramatic apoptosis of HCC and CRC cells rather than normal cells induced by PS341 with Hoechst33342 staining in which the white arrow indicated the cells undergoing DNA fragmentation and apoptosis (Fig. 1c). In the DNA ladder experiments, HCC and CRC cells displayed the remarkable DNA fragments than the normal cell lines L02 and FHC (Fig. 1d, left panel). The percent of apoptosis cells was estimated by a colorimetric assay to measure the amount of DNA fragmentation induced by PS341. PS341 significantly stimulated the apoptosis of HCC and CRC cells rather than normal cell lines L02 and FHC by increase in absorbance corresponding to the increase in apoptotic DNA fragments (Fig. 1d, right panel). Collectively, these *in vitro* data indicated PS341 could induce specific apoptosis and suppression of proliferation of HCC and CRC cells.

### PS341 downregulates the migration and invasion capabilities of HCC and CRC cells

**Figure 2:**
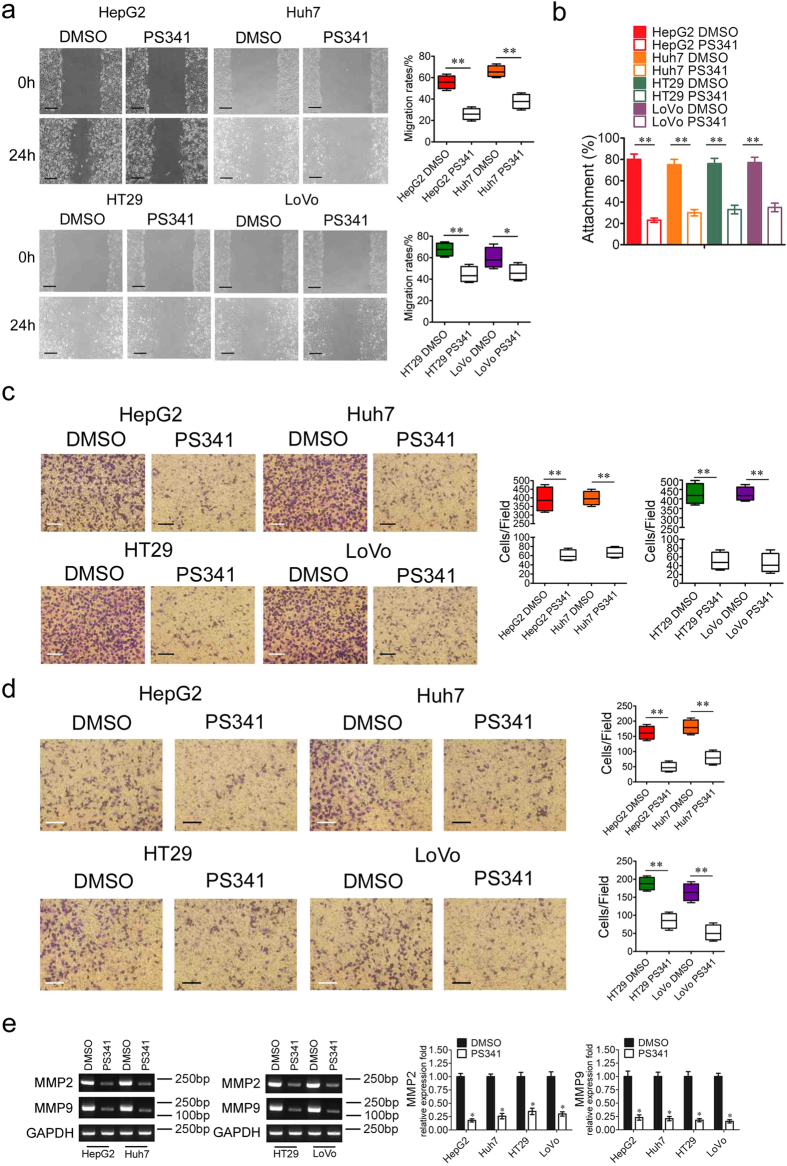
PS341 downregulates the migration and invasion capabilities of HCC and CRC cells. (**a**) PS341 reduces migration of HCC and CRC cells in wound-healing assays. DMSO or PS341 were added into the medium of HepG2, Huh7, HT29 and LoVo cells after the artificial wound. Migration rates were calculated by healing area/wound area 24 h later. Scale bar = 50  μm. (**b**) PS341 negatively regulates the adhesion of HCC and CRC cells to laminin. (**c**) PS341 significantly suppresses the migration of cancer cell lines with transwell analysis. Scale bar = 25 μm. (**d**) PS341 significantly inhibits the invasion of cancer cell lines with boyden chamber analysis. Scale bar = 25 μm. (**e**) PS341 decreases expression of MMP2 and MMP9 in HCC and CRC cells in the PCR and RT-PCR analysis. The full-length gels are presented in Supplementary Fig. 1. The whole assay was repeated three times. Data are presented as mean ± SD. *P < 0.05, **P < 0.01.

Migration and metastasis are hallmarks of cancer development^14^ and we further detected the function of PS341 in the migration of HCC and CRC cells. The migration of cultured HCC and CRC cells were significantly inhibited by PS341 in the wound-healing process after scratch (Fig. 2a). And in the cell adhesion assay, the cells were plated in the laminin coated wells and incubated at 37 °C for 2 h with/without 10 nM PS341. Adherent cells were fixed with 4% formaldehyde and stained with crystal violet. We also found PS341 could greatly decrease the cell attachment to laminin in both HCC and CRC cells (Fig. 2b). In consistent with the data in wound-healing assay, PS341 could also very effectively suppress the migration of HCC and CRC cells in transwell assays (Fig. 2c). With the treatment of PS341, the ability of invasion through matrigel of HepG2, Huh7, HT29 and LoVo cancer cells also significantly decreased (Fig. 2d). As type IV collagenases, matrix metalloproteinase 2 (MMP2) and MMP9 are activated in many tumors and are associated with the increased transfer capacity for these tumors^15^. Our data also indicated that PS341 treatment largely decreased the expression of both MMP2 and MMP9 in HCC and CRC cells (Fig. 2e), thus restricting the migration capacities of these two tumor types. Taken together, PS341 could effectively suppress the migration and invasion capabilities of HCC and CRC cells *in vitro*.

### PS341 suppresses the epithelial-mesenchymal transition of HCC and CRC cells

**Figure 3:**
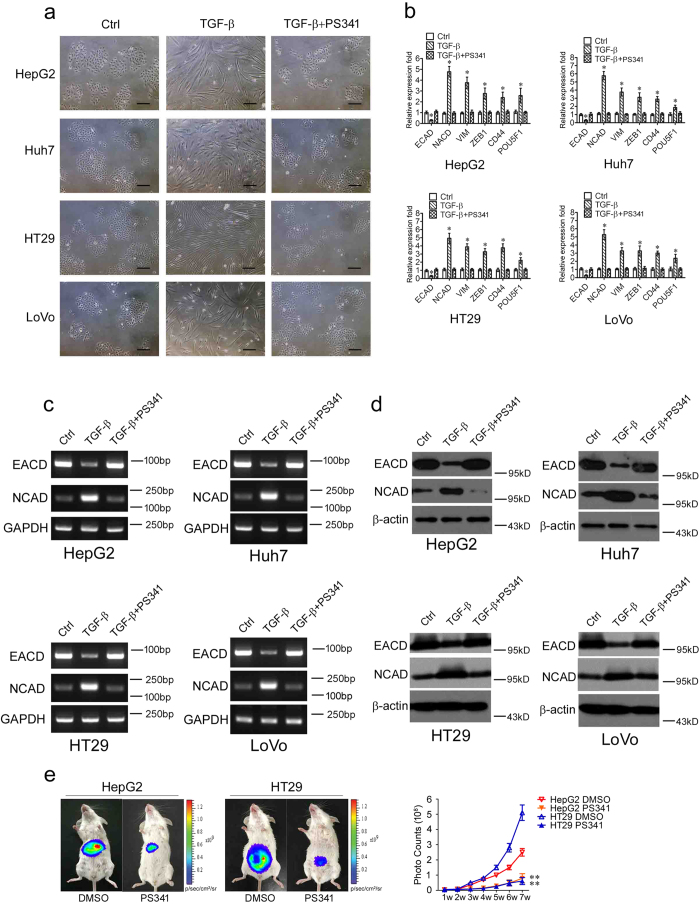
PS341 suppresses the epithelial-mesenchymal transition of HCC and CRC cells. (**a**) PS341 reverses the EMT transition induced by TGF-β1 in HCC and CRC cells. Scale bar = 25 μm. (**b**) The RT-PCR analysis of the mRNA expression of ECAD, NCAD, VIM, ZEB1 CD44 and POU5F1 in HCC and CRC cells with TGF-β1 and PS341 treatment. (**c**) The PCR analysis of the mRNA expression of the epithelial marker E-cadherin and the mesenchymal marker N-cadherin was carried out in HCC and CRC cells with TGF-β1 and PS341 treatment. (**d**) Protein expression levels of E-cadherin and N-cadherin were carried out in HCC and CRC cells with TGF-β1 and PS341 treatment. (**e**) PS341 suppresses the proliferation and metastasis of HepG2 and HT29 cancer cells in the peritoneal spreading mice models. 2 × 10^6^ luciferase-expressing HepG2 and HT29 cancer cells were injected intraperitoneally into NOD/SCID mice with the administration of PS341 or DMSO twice a week, and the tumor cells were monitored once a week (n = 5). The whole assay was repeated three times. Data are displayed as mean ± SD. TGF-β: TGF-β1. The full-length blots/gels are presented in Supplementary Figs 1–3. *P < 0.05, **P < 0.01.

The epithelial-mesenchymal transition (EMT) is a key process for epithelial cells to harbor mesenchymal properties and is closely engaged in cancer invasion and metastasis^16^. To evaluate the effect of PS341 on this process, we induced EMT of HCC and CRC cells with TGF-β1^16^,^17^ and found the cell lines showed mesenchymal appearance, which was fibroblast-like (Fig. 3a, middle). When added with PS341 in the treatment, however, the cancer cell lines remained epithelial appearance, indicating that PS341 efficiently blocked EMT induced by TGF-β1 (Fig. 3a, right).

To further determine the inhibition of EMT by PS341 at the molecular levels, we detected the mRNA expression levels of EMT markers including E-cadherin (ECAD), N-cadherin (NCAD), Vimentin (VIM), ZEB1, CD44 and POU5F1 with the treatment of TGF-β1. In the real-time polymerase chain reaction (RT-PCR) analysis, HCC and CRC cells underwent EMT with the decreased expression of epithelial cell marker ECAD^18^ and the increased expression of mesenchymal cell marker NCAD, VIM, ZEB1, CD44 and POU5F1 significantly^16^,^19^ (Fig. 3b). However, when treated with PS341 combined with TGF-β1, HCC and CRC cells regained the epithelial cell characteristics and kept the same expression levels of EMT markers as control cells (Fig. 3b). These results indicated that PS341 could effectively inhibit the epithelial-mesenchymal transition of HCC and CRC cells.

We selected epithelial cell marker ECAD and mesenchymal cell marker NCAD two significantly altered markers for further confirmation. In the PCR assay, PS341 suppressed the EMT of HCC and CRC cells by upregulating the expression of ECAD and downregulating the expression of NCAD, in which GAPDH was as the loading control (Fig. 3c). Similarly, PS341 abrogated the EMT of HCC and CRC cells by upregulating the expression of ECAD and downregulating the expression of NCAD in the protein expression levels, in which β-actin was as the loading control (Fig. 3d). Given that PS341 inhibits migration and EMT of HCC and CRC cells *in vitro*, we next verified whether PS341 could suppress the metastasis of HCC and CRC cells *in vivo*. pGL3 overexpression HepG2 and HT29 cancer cells were applied in the peritoneal spreading mice models. 2 × 10^6^ HepG2 and HT29 cells were intraperitoneally injected into NOD/SCID mice, and 0.5 mg/kg PS341 was administered twice per week till seven weeks by intraperitoneal injections. Tumor growth was monitored by bioluminescence imaging using the Xenogen IVIS Lumina system (Xenogen Corporation, Hopkinton, MA) every week (Fig. 3e). These data indicated that PS341 markedly suppressed the growth and metastasis of HepG2 and HT29 cells *in vivo* (Fig. 3e). Collectively, PS341 not only efficiently inhibited the migration and EMT of HCC and CRC cells *in vitro,* but also significantly suppressed the propagation and metastasis of HepG2 and HT29 cancer cells *in vivo*.

### PS341 induces cell cycle arrest in HCC and CRC cells

**Figure 4:**
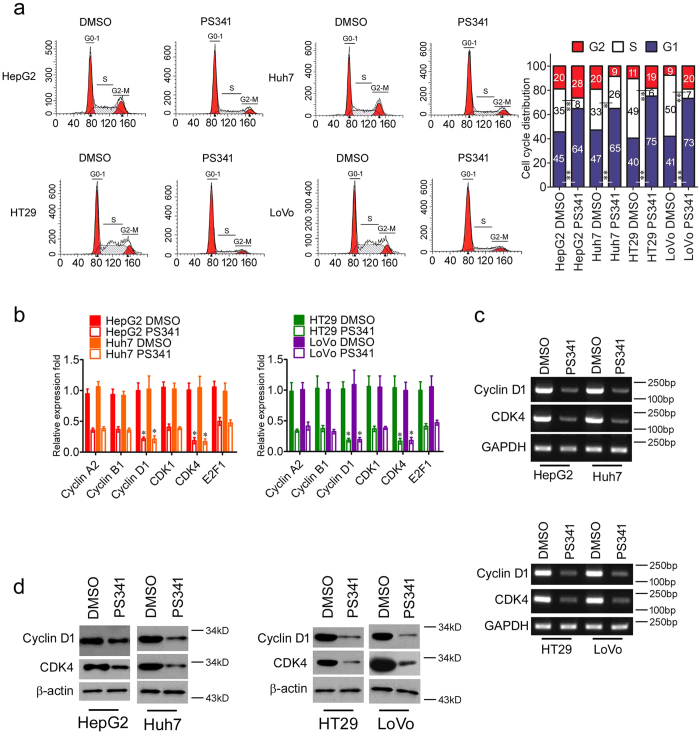
PS341 induces cell cycle arrest in HCC and CRC cells. (**a**) PS341 induces G0/G1 cell cycle arrest in both HCC and CRC cells. DMSO or PS341 were supplemented into the medium of HepG2, Huh7, HT29 and LoVo cells and cell cycle analysis was processed after 70% ethanol fixation and PI staining. (**b**) The RT-PCR analysis of the mRNA expression levels of Cyclin A2, Cyclin B1, Cyclin D1, CDK1, CDK4 and E2F1 in HCC and CRC cells with PS341 treatment. (**c**) PS341 inhibits the expression of Cyclin D1 and CDK4 at transcriptional level in HCC and CRC cells. (**d**) PS341 suppresses the expression of Cyclin D1 and CDK4 at the protein level in HCC and CRC cells. The whole assay was repeated three times. The full-length blots/gels are presented in Supplementary Figs 3 and 4. Data are displayed as mean ± SD. *P < 0.05, **P < 0.01.

Insensitivity to anti-growth signals is another hallmark of cancer and there is aberrant regulation of cell cycle in tumor cells^14^. Cell cycle analysis showed that both HCC and CRC cells were mostly arrested within the G0/G1 phase, indicating there was a reduced number of dividing tumor cells with PS341 treatment (Fig. 4a). There were also fewer cells under S phase, showing that PS341 could inhibit DNA replication too (Fig. 4a).

Cyclin D1/CDK4 complex is required for transition from G1 phase to S phase^20^, so we next checked the expression levels of Cyclin D1, CDK4 and other cell cycle related markers such as Cyclin A2, Cyclin B1, CDK1 and E2F1. In the RT-PCR analysis, we found PS341 suppressed the expression of Cyclin A2, Cyclin B1, Cyclin D1, CDK1, CDK4 and E2F1 in the mRNA levels in HCC and CRC cells, in which the expression of Cyclin D1 and CDK4 were remarkably inhibited (Fig. 4b,c). Meanwhile, the protein expression levels of Cyclin D1 and CDK4 were also decreased in HCC and CRC cells under the treatment of PS341 (Fig. 4d). Taken together, PS341 could negatively regulate the expression of Cyclin D1/CDK4 and thus induce the G0/G1 arrest in both HCC and CRC cells.

### PS341 reduces the expression of stemness-related genes in HCC and CRC cells

**Figure 5:**
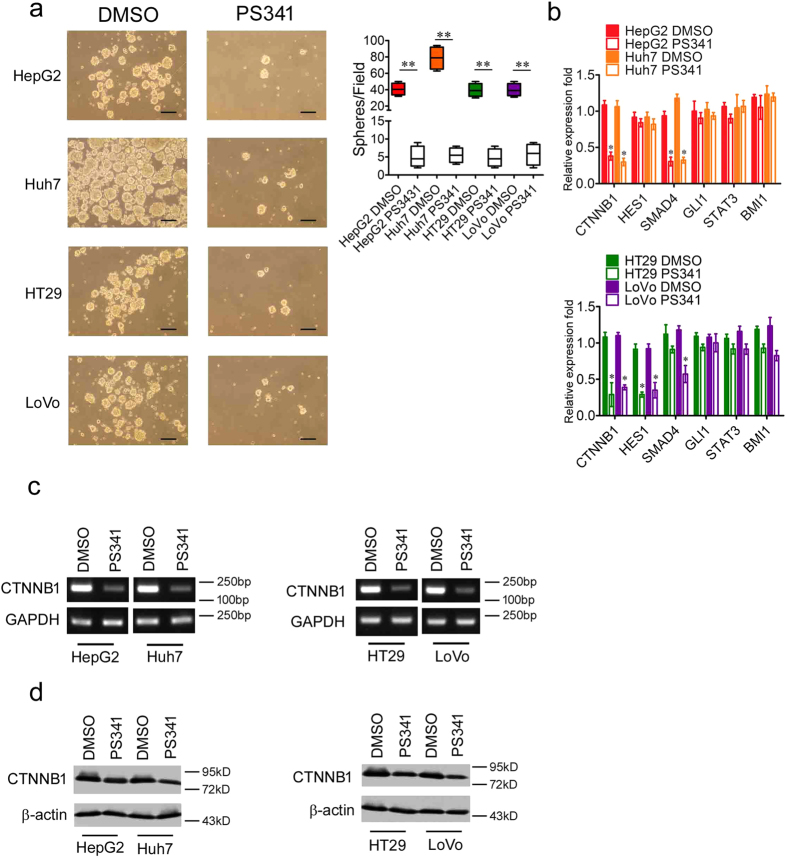
PS341 reduces the expression of stemness-related genes in HCC and CRC cells. (**a**) PS341 significantly suppresses the formation of tumor spheres of HCC and CRC cells. Scale bar = 50 μm. (**b**) mRNA expression levels of some key stemness-related genes in HCC and CRC cells with PS341 treatment. (**c**) PS341 greatly suppresses the expression level of CTNNB1 at the transcriptional level. (**d**) PS341 greatly inhibits the expression level of CTNNB1 at the protein level. The whole assay was repeated three times. The full-length blots/gels are presented in Supplementary Figs 4 and 5. Data are displayed as mean ± SD. *P < 0.05, **P < 0.01.

Cancer stem cells or tumor initiating cells are a small portion of cancer cells and they harbor the stem cell-like characteristics, which can give rise to all kinds of cancer cells within a specific tumor^21^. Unlike the normal cancer cells, the cancer stem cells can maintain their stemness through higher expression of some stem cell regulators and are more resistant to anti-cancer drugs^21^. Sphere-initiating capacity is one of the important features of cancer stem cells^21^. In order to explore the function of PS341 in HCC and CRC stem cells, we employed cell lines of both cancers in sphere formation assays. The results indicated PS341 significantly reduced sphere genesis in both HCC and CRC cells (Fig. 5a).

We further detected the expression of some reported stemness-related genes in HCC and CRC stem cells including *CTNNB1*, *HES1*, *SMAD4*, *GLI1*, *STAT3* and *BMI1*^22^,^23^,^24^,^25^ in the cancer cell lines with or without PS341 treatment. In the RT-PCR analysis, we found that in both of the two liver cancer cell lines, the expression of CTNNB1 and SMAD4 was greatly inhibited by PS341 while the other four stemness-related genes remained almost the same (Fig. 5b, top). As for the colon cell lines, the expression of CTNNB1 and HES1 were suppressed significantly in both HT29 and LoVo by PS341 and SMAD4 expression was only reduced greatly in LoVo but not HT29 (Fig. 5b, bottom). These data showed PS341 could inhibit some specific stemness-related genes which are dependent on tumor types.

Among the stemness-related genes we checked, only *CTNNB1* was inhibited by PS341 all across the cell lines originated from both HCC and CRC. We confirmed this data with PCR assay, in which PS341 significantly suppressed the mRNA expression levels of CTNNB1 in both HCC and CRC cells (Fig. 5c). In consistent with the transcriptional level, the protein expression of CTNNB1 was also inhibited efficiently by PS341 treatment in all the four cell lines (Fig. 5d). All these data indicated an essential role of CTNNB1 in the development of HCC and CRC, providing a potential target for clinical treatment.

### PS341 upregulates the expression of FOXO3 and inhibits the transcription of *CTNNB1*

**Figure 6:**
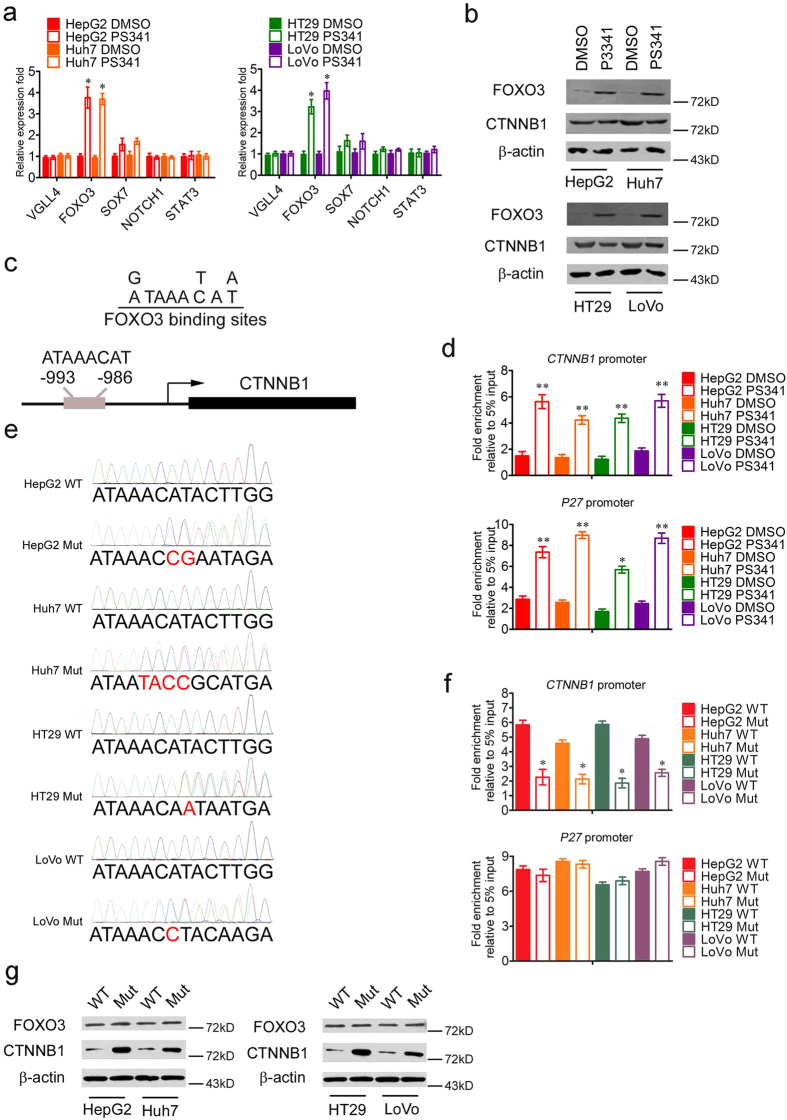
PS341 upregulates the expression of FOXO3 and inhibits the transcription of *CTNNB1*. (**a**) The RT-PCR analysis of the mRNA expression of VGLL4, FOXO3, SOX7, NOTCH1 and STAT3 in HCC and CRC cells with PS341 treatment. (**b**) PS341 decreases the degradation of FOXO3 and inhibits the transcription of *CTNNB1*. The full-length blots are presented in Supplementary Fig. 5. (**c**) Recognition sequence of FOXO3 in *CTNNB1* promoter. (**d**) Chromatin immunoprecipitation analysis of *CTNNB1* and *p27* promoter with IgG and FOXO3 antibodies in HCC and CRC cells with/without PS341 treatment. (**e**) Sanger sequencing of PCR product including *CTNNB1* promoter in HepG2 WT, HepG2 Mut, Huh7 WT, Huh7 Mut, HT29 WT, HT29 Mut, LoVo WT and LoVo Mut cells. Red bases indicated the abrogation of the binding motif of FOXO3. (**f**) Chromatin immunoprecipitation analysis of *CTNNB1* and *p27* promoter with IgG and FOXO3 antibodies in HepG2 WT, HepG2 Mut, Huh7 WT, Huh7 Mut, HT29 WT, HT29 Mut, LoVo WT and LoVo Mut cells with PS341 treatment. (**g**) Western blot analysis of FOXO3 and CTNNB1 in HepG2 WT, HepG2 Mut, Huh7 WT, Huh7 Mut, HT29 WT, HT29 Mut, LoVo WT and LoVo Mut cells with PS341 treatment. The deletion of the binding sequence of FOXO3 in *CTNNB1* promoter relieved the transcriptional inhibition of *CTNNB1*. The full-length blots are presented in Supplementary Fig. 6. Data are displayed as mean ± SD. *P < 0.05, **P < 0.01.

In order to decipher the mechanisms of the downregulation of CTNNB1 in HCC and CRC cells under the treatment of PS341, we focused on the negative transcriptional regulators of CTNNB1 such as VGLL4, FOXO3, SOX7, NOTCH1 and STAT3^26^,^27^,^28^,^29^,^30^,^31^. In the RT-PCR analysis, only the mRNA expression level of FOXO3 significantly increased in both HCC and CRC cells after the treatment of PS341 (Fig. 6a). *FOXO3* is a member of the forkhead family of transcription factors and could be activated by the stimulus of growth factors and cellular stress, which functions as a transcription factor/coactivator to trigger the expression of target genes for apoptosis and other physiological processes^32^,^33^. In the western blot analysis, the protein expression levels of FOXO3 was also increased in HCC and CRC cells under the treatment of PS341 with the protein expression levels of CTNNB1 downregulation (Fig. 6b).

According to the previous studies, FOXO3 could directly interact with CTNNB1 and suppressed the transcription of CTNNB1/TCF complex, or FOXO3 activated the expression of microRNA-34b/c which inhibited the mRNA expression of CTNNB1 by occupying its untranslated regions (UTRs)^29^. Whether there is a direct transcriptional regulation of CTNNB1 by FOXO3 remains unknown. We first analyzed the promoter sequence of *CTNNB1* ranging from −2000 ~ −1 and looked for the specific binding sites of FOXO3 (Fig. 6c). Interestingly, we found a distinctive binding sequence ATAAACAT of FOXO3 in the promoter of *CTNNB1* ranging from −993 ~ −986 (Fig. 6c).

Then we carried out the chromatin immunoprecipitation (CHIP) experiments in HCC and CRC cells by IgG and FOXO3 antibodies. After the CHIP and RT-PCR analysis, we found that FOXO3 indeed occupied the ATAAACAT sequence of *CTNNB1* promoter in HCC and CRC cells, in which *p27* is the known target of FOXO3 as the positive control. The results indicated FOXO3 was significantly enriched on the promoters of *CTNNB1* and *p27* after the treatment of PS341 in HCC and CRC cells mainly resulting from the elevated expression of FOXO3 (Fig. 6d).

In order to provide direct evidence with respect to FOXO3 regulating CTNNB1 in the context of PS341, we applied the CRISPR-Cas9 technology to obtain the liver and colon cell lines in which the binding sequence of FOXO3 in *CTNNB1* promoter is deleted (named HepG2/Huh7/HT29/LoVo Mut). The deletion of DNA sequence was confirmed by DNA Sanger sequencing (Fig. 6e). Then in the CHIP experiments by FOXO3 antibody, FOXO3 significantly occupied on the promoter of *CTNNB1* in HCC and CRC wild type (WT) cells under the treatment of PS341 (Fig. 6f, top). However, the abrogation of ATAAACAT motif in the promoter of *CTNNB1* remarkably inhibited the binding of FOXO3 to *CTNNB1*′s promoter in HCC and CRC Mut cells (Fig. 6f, top). Nevertheless, the removal of ATAAACAT motif in the promoter of *CTNNB1* had no influence on the binding of FOXO3 to the promoter of *P27* (Fig. 6f, bottom). Furthermore, we test the protein expression levels of FOXO3 and CTNNB1 in WT and Mut liver and colon cell lines. The western blot results indicated that the deletion of the binding sequence of FOXO3 in *CTNNB1* promoter relieved the transcriptional inhibition of *CTNNB1* mediated by FOXO3 and upregulated the expression of CTNNB1 in HCC and CRC Mut cells compared with that of HCC and CRC WT cells (Fig. 6g). Meanwhile, the expression of FOXO3 remained unchanged between former two groups (Fig. 6g). Taken together, PS341 enhanced the expression of FOXO3 in mRNA and protein levels in HCC and CRC cells, in which FOXO3 could bind to the promoter of *CTNNB1* and suppressed the expression of CTNNB1.

Whether PS341 affects FOXO3 binding and CTNNB1 expression in normal cells? First we analyze the expression levels of FOXO3 and CTNNB1 in normal cell lines (L02 and FHC) and cancerous cell lines (HepG2, Huh7, HT29 and LoVo). The RT-PCR results suggested that FOXO3 was highly downregulated in tumor cells, yet CTNNB1 was remarkable upregulated in tumor cell (Supplementary Fig. 7a). These data was consistent with previous studies^34^,^35^. Second, the mRNA expression level of FOXO3 slightly increased in L02 and FHC cells after the treatment of PS341, yet the mRNA expression level of CTNNB1 remained unchanged (Supplementary Fig. 7b). The stable expression of CTNNB1 indicated that, on one hand, the original expression level of FOXO3 was already high and the elevated amount of FOXO3 caused by PS341 had little effect on the inhibition of *CTNNB1* transcription. On the other hand, the transcriptional regulation of *CTNNB1* in normal cells was controlled by some other factors and different from that of tumor cells. Finally, PS341 treatment slightly augmented the binding ability of FOXO3 to the *CTNNB1*′s promoter sequence resulted from the enhanced expression of FOXO3 (Supplementary Fig. 7c). Taken together, PS341 had little effect on the FOXO3 binding and CTNNB1 expression in normal cells. However, the specific mechanisms of *CTNNB1* transcriptional regulation in normal cells need to be further investigated.

### PS341 promotes the survival of mice bearing HCC or CRC

**Figure 7:**
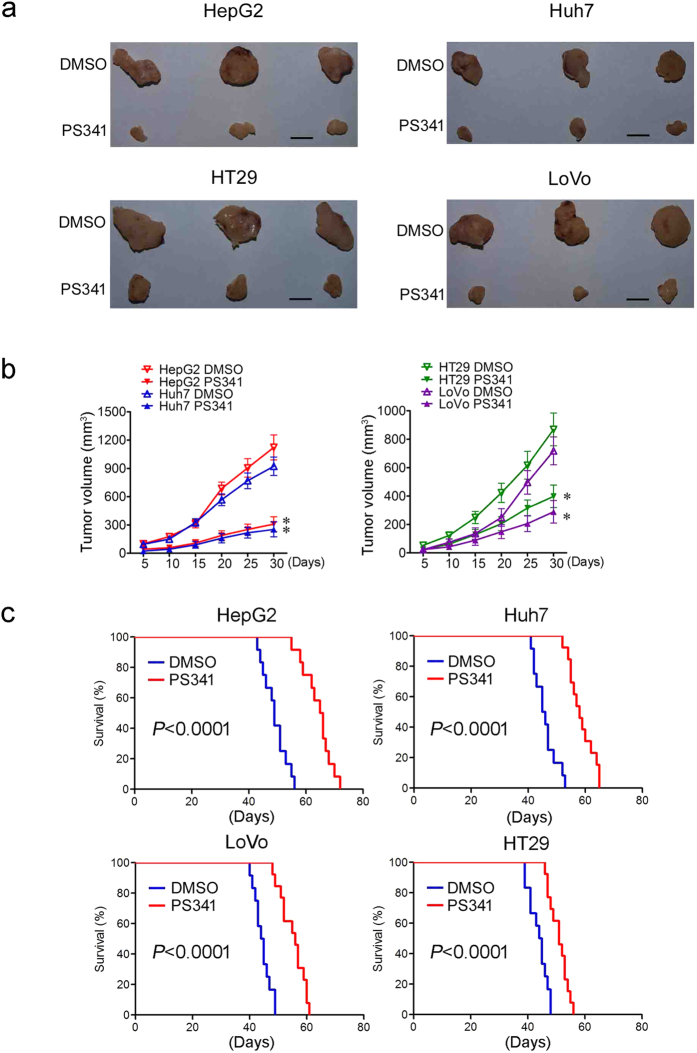
PS341 promotes the survival of mice bearing HCC or CRC. (**a,b**) PS341 suppresses tumor growth of HCC and CRC cells in xenograft mice. Mice bearing tumor cells of HepG2, Huh7, HT29 and LoVo were administered intraperitoneally with DMSO or PS341 two times per week. The volume of xenografts was measured every four days. The tumors were removed after the mice sacrificed 30 days later. Data are expressed as mean ± SD. *P < 0.05. Scale bar = 50 mm. (**c**) PS341 augments the survival of xenograft mice transplanted with different HCC and CRC cells.

Xenograft cancer models in mice were thus employed and HCC or CRC cells were injected subcutaneously into the back of nude mice respectively. Three or four days later, cancer xenografts were found on mice. On day five after tumor implantation, 0.5 mg/kg PS341 was injected intraperitoneally and then administrated twice per week till day 30 when the mice were sacrificed and the therapeutic effect was evaluated. In consistence with the *in vitro* data, the size (Fig. 7a) and volume (Fig. 7b) of both HCC and CRC were significantly reduced by PS341 administration compared with the control groups.

In order to further clarify the protective role of PS341 *in vivo*, the survival rate of mice bearing HCC or CRC cells were analyzed. It was shown that PS341 significantly augmented the lifespan of mice which were transplanted with all tested HCC or CRC cells (Fig. 7c). Taken together, PS341 could inhibit the tumor development, reduce the tumor burden of HCC and CRC cells in the xenograft model and effectively extend the lifespan of mice bearing cancer cells.

## Discussion

The precise control of protein degradation by ubiquitin-proteasome system is critical for signal transduction, transcriptional regulation, response to stress, and receptor function^36^,^37^. In cancers, dysregulation of this catalytic process may contribute to tumor progression, drug resistance, and altered immune surveillance^38^. Proteasome inhibition can affect the levels of various short-lived proteins, and result in inhibition of NF-кB, increased activity of p53 and Bax proteins, or accumulation of cyclin dependent kinase inhibitors p27 and p21, thus leading to apoptosis. Preclinical studies showed that malignant, transformed, and proliferating cells were more susceptible to proteasome inhibition than normal cells, making it a useful target in cancer therapy^38^,^39^.

PS341 is a boronic acid dipeptide, which is a specific inhibitor of the proteasome machine^9^,^40^. It can inhibit the proteasome pathway rapidly in a reversible manner by binding directly with the 20S proteasome complex and blocking its enzymatic activity^9^,^40^.

The hepatocellular carcinoma (HCC) and colorectal cancer (CRC) are both most common malignant tumors, the treatment of which is now very limited. The molecular targeted therapy with its high-efficiency and low toxicity completely changed the conventional tumor treatment^41^. It launched a revolution in the cancer therapy and became a largest hotspot of cancer research in recent years. The successful development of Sorafenib was promising in drug therapy for advanced HCC, but its effectiveness is still not satisfied^4^,^42^. In order to overcome the present dilemma of HCC and CRC therapy, the mechanism of pathogenesis and progression of the two tumor types needs to be clearly studied and more specific targeted drugs have to be exploited.

In our early study, we found the ubiquitin cross-reactive protein ISG15 is associated with the malignant behavior of liver cancer, so we subsequently speculated the ubiquitin proteasome pathway should be abnormally activated in HCC^43^. Taken the fact into account that PS341 either alone or in combination had unsatisfactory clinical activities in phase II studies in patients with hepatocellular carcinoma or colorectal cancer^12^,^13^, we got the inspiration to explore the mechanisms of PS341 in HCC and CRC treatment and reveal potential therapeutic strategies combined with PS341 towards HCC and CRC.

In this study we found that PS341 at a low concentration (10 nM) effectively inhibited the growth of HCC and CRC cells and suppressed their migration and invasion abilities. Epithelial-mesenchymal transition, which is critical in tumor metastasis, was also efficiently inhibited by PS341 treatment. PS341 was also proved to cause cell cycle arrest and apoptosis of HCC and CRC cells at a low concentration (10 nM). Moreover, it abrogated the sphere formation and self-renewal capabilities of HCC and CRC cells with the downregulation of key transcription factor CTNNB1. The molecular mechanisms of PS341 on apoptosis promotion, cell cycle arrest, inhibition of migration, invasion, EMT, self-renewal capabilities were the upregulation of FOXO3, in which FOXO3 bound to the promoter sequence ATAAACAT of *CTNNB1* ranging from −993 ~ −986 and inhibited the transcriptional activation of *CTNNB1*.

These results indicated HCC and CRC cells are highly sensitive to PS341. Moreover, we also checked many other tumors and found esophagus cancer, gastric cancer and pancreas cancer cells were not so sensitive to PS341 (data not shown), indicating that PS341 is more promising in treatment of specific cancers such as HCC and CRC. This difference among cancer types may result from the specific regulation of tumorigenesis by ubiquitin-proteasome system in an individual cancer, the mechanism of which is of great interest to be further investigated.

In mouse models, we found the commonly used dosage of PS341 (1.0 mg/kg twice per week via tail vein) as described before^44^ caused huge toxicity and mice could develop apparent anorexia, weight lose, severe anemia and bleeding, leading to high mortality. Therefore we halved the dose to 0.5 mg/kg twice per week and found decreased dosage could also make obvious inhibition of tumor growth and extension the lifespan of mice bearing cancer cells.

Our data demonstrated PS341 could inhibit the growth of HCC and CRC and induce the apoptosis both *in vitro* and in the xenograft mouse models. Our research discovered PS341 enhanced the expression of FOXO3, which occupied the promoter of stemness-related gene *CTNNB1* and suppressed the expression of it. These results indicated that the downregulation of CTNNB1 played a critical role in HCC and CRC therapy and explained the possible reasons for the unsatisfactory clinical activities in phase II studies of PS341. It may result from the dose and the schedule of the treatment, or the inadequate inhibition of CTNNB1 and ubiquitin-proteasome pathway. Thus, in order to make PS341 more effective in future clinical application in HCC and CRC therapy, firstly, in combination with other approaches should be encouraged to be investigated, including the suppression of CTNNB1 which was discovered as the therapeutic target in this paper. Additionally, upgrade and development of next generation proteasomes inhibitors based on the backbone of PS341 may help to reduce the side effects and improve the specificity and efficacy to HCC and CRC patients. Finally, the dose, the schedule and the delivery method of the treatment still have some room for improvement. For example, the drug delivery system should be optimized to make sure that PS341 could be effectively released in specific tumor cells with high pharmaceutical activity rather than inactivated by some natural compounds.

In conclusion, our current research proposed that PS341 could effectively inhibit cell proliferation, EMT, cell migration and invasiveness and stem cell properties of HCC and CRC both *in vitro* and *in vivo* by regulating the FOXO3/CTNNB1 axis. Moreover, our study underscored that CTNNB1 may be a promising target for combination therapy with PS341in HCC and CRC clinical treatment. All these data laid some basis for the future clinical application of PS341 combined with suppression of CTNNB1 in HCC and CRC therapy and further investigation on the development of novel and effective therapeutic strategies.

## Methods

### Reagent and antibodies

Antibodies that recognize Cyclin D1 (Cell Signaling Technology, #2922), CDK4 (Cell Signaling Technology, #12790), β-actin (Sigma, A1978), NCAD (Abcam, ab98952), ECAD (Cell Signaling Technology, #3195), FOXO3 (Abcam, ab12162) and CTNNB1 (Ser33/37/Thr41) (Cell Signaling Technology, #4270) were used in the study. Horseradish peroxidase (HRP) labelled secondary antibody (Beyotime Biotech) was used in western blots. PS341 was purchased from Santa Cruz, sc-217785.

### Cell line

Cell lines HepG2, Huh7, FHC, HT29, LoVo SW620, SW480 and LS180 were obtained from American Type Culture Collection (Rockville, MD, US). Cell lines L02, 97L, 97H and M3 were purchased from Liver Cancer Institute, Zhongshan Hospital affiliated to Fudan University (Shanghai, china)^45^.

### Cell proliferation assay

The proliferation and viabilities of the cells were measured by the cell counting kit-8 (CCK-8; Dojindo, Kumamoto, Japan) colorimetric assay with triplicate experiments for each set of conditions. The same amount of cells (3 × 10^3^ cells per well) were seeded on 96-well culture plates and cultured with/without 5 nM, 10 nM or 20 nM PS341 for 24 h, 48 h and 72 h. At the indicated time points, the supernatant was removed and 100 μl of DMEM medium containing 10 μl of CCK8 was added to each well for 2 h at 37 °C. The absorbance at 450 nm was measured with a plate reader (Multiskan GO Microplate Spectrophotometer; Thermo Fisher Scientific, Inc., Waltham, MA, USA). The experiments were repeated three times.

### Cellular apoptosis assay

The cells were cultured with 10 nM PS341 for 48 h, and apoptosis was assessed with the Annexin V-FITC kit according to the manufacturer’s instructions. The cells were washed twice with cold PBS, digested, collected, and resuspended in binding buffer. Annexin V-FITC and PI were added (BioVision, Milpitas, CA, USA), and the cells were incubated for 10 min at room temperature in the darkness. Then 200 μl binding buffer was added, and the Annexin V positive cells were analyzed using a FACSCalibur flow cytometry system (BD Biosciences, US). The experiments were repeated three times.

### DNA fragmentation experiment

The cells were cultured with 10 nM PS341 for 48 h, collected by centrifugation and washed two times with PBS. In the Hoechst staining assay, the cells were mixed and incubated with the Hoechst 33258 dye (1 μg/ml; Sigma) for 10 min. The fluorescence of the apoptotic cells is determined with a UV-equipped fluorescence microscope. The number of apoptotic cells is counted in five independent fields for each cell type.

While in the DNA ladder assay, the cell pellets were lysed for 30 s with lysis buffer (1% NP-40 in 20 mM EDTA, 50 mM Tris-HCl, pH 7.5; 100 μl per 10^6^ cells). The supernatant was collected at 1600g for 5 min centrifugation and repeated with lysis buffer once. Then 1% SDS and 5 μg/ml RNase A were added into the supernatant at 56 °C for 2 h, and 2.5 μg/ml proteinase K was added into the solution 37 °C for 2 h. 1/2 volume of 10 M ammonium acetate was added into the solution followed by 2.5 volume of ethanol to precipitate DNA. The extraction of DNA is dissolved in loading buffer for electrophoresis analysis.

### Wound healing assay

2×10^5^ HCC and CRC cells were seeded into a 24 well plate. The tumor cells grew to confluence 24 h later. An artificial wound was introduced with a P10 pipette tip per well. Data of the wounded area were taken at 0 h and 24 h with a microscope (Olympus Corp). The whole assay was repeated four times.

### Cell adhesion and inhibition assay

Cell adhesion assays using HepG2, Huh7, HT29, LoVo cells and laminin were performed as described previously^46^. In short, 96-well plates (NEST) were incubated with laminin at 4 °C overnight and then blocked with PBS containing 1% BSA for 1 h at 37 °C. The cells were plated on the coated wells and incubated at 37 °C for 2 h with/without 10 nM PS341. Adherent cells were fixed with 4% formaldehyde and stained with crystal violet. The stained cells were counted under a microscope in five fields per well.

### Transwell assay

HepG2, Huh7, HT29 and LoVo cells were respectively harvested, washed, suspended with DMEM (GIBCO, Life Technologies, 11965-092) and seeded to the upper chambers of transwell inserts (8 μm pore size; Corning) with/without 10 nM PS341 in the migration assay. What’s more, the upper chambers were coated with Matrigel (BD Bioscience, 354234) before the inoculation of the cancer cells and PS341 in the invasion assay.

The lower compartments were filled with DMEM supplemented with 5% FBS (fetal bovine serum). The cells in the upper chamber were removed with a swab after incubation for 12 h in the migration assay or 24 h in the invasion assay. The cells that migrated to the lower layer and attached to the membrane were stained with crystal violet and numbered in five fields per well under a microscope. The whole assay was repeated three times.

### PCR and Real-Time PCR

Total RNA from cells was extracted using an RNA isolation kit (Qiagen) and subjected to cDNA synthesis using M-MLV Reverse Transcriptase (Promega). The cDNA were then used as the templates for semi-quantitive PCR and quantitative analysis of the candidate genes, running in an ABI 7300 analyzer (Applied Biosystems). Primer sequences are listed in Supplementary Table 1. SYBR Green (Qiagen) was used as the fluorescent probe. Relative expression levels of the target genes were compared to a housekeeping gene, *GAPDH*.

### Epithelial-mesenchymal transition (EMT) induction

HepG2, Huh7, HT29 and LoVo cells were cultured to attach in the complete medium overnight. Then the cells were maintained in either medium alone or medium supplemented with 2.5 ng/ml TGF-β1 (R&D Systems, Minneapolis, MN), with or without 10 nM PS341 in a humidified 5% CO2 incubator at 37 °C. The morphologic photos of the cells were taken seven days after the incubation. The experiments were repeated three times.

### Western blot

HepG2, Huh7, HT29 and LoVo cells were lysed with ice-cold RIPA buffer (50 mM Tris-HCl [pH 7.4], 150 mM NaCl, 0.5% sodium desoxycholate, 0.1% SDS, 5 mM EDTA, 2 mM PMSF, and 1% Nonidet P-40) for 2 h. Proteins were separated with polyacrylamide gel electrophoresis and transferred to a nitrocellulose membrane (Millipore). The membranes were blocked with skim milk, probed with primary antibodies and HRP-conjugated secondary antibodies and developing with Pierce™ ECL Western Blotting Substrate (Thermo Scientific™)^47^.

### Cell cycle assay

HepG2, Huh7, HT29 and LoVo cells were cultured with/without 10 nM PS341 for 48 h, digested and collected by trypsin, fixed in 70% ethanol overnight at 4 °C and then stained with 50 μg/ml propidium iodide (PI) (Sigma, St. Louis, MO, USA) and 0.1 μg/ml RNase A (Sigma, St. Louis, MO, USA). The cells were analyzed using a FACSCalibur flow cytometry system (BD Biosciences, US). CellQuest software (BD CellQuest Pro Software, BD Biosciences, US) was used to analyze the percentage of the cell population in each phase.

### Sphere formation

5×10^3^ cells of HepG2, Huh7, HT29 and LoVo were seeded in an ultra*-*low attachment surface 6*-*well plate (Corning). Cells were maintained in DMEM/F12 (Gibco) medium supplemented with 20 ng/mL EGF (Sigma), 20 ng/mL bFGF (Sigma), 1% N2 (Gibco), 2% B27 (Gibco). The number of spheres was counted 14 days after seeding. The whole assay was repeated four times.

### Chromatin immunoprecipitation (ChIP)

Chromatin immunoprecipitation was performed by using the ChIP assay kit according to the manufacturer’s instructions (Upstate Biotechnology). Briefly, approximately 10 million cancer cells were crosslinked with 1% formaldehyde for 10 min at 37 °C, washed with PBS, and resuspended in lysis buffer. The cell lysate was sonicated on ice for a total of 2 min (in 5 s pulses), resulting in an average DNA fragment length of 500 bp. After removing cell debris by centrifugation and pre-clearing the lysate, immunoprecipitation was performed in ChIP dilution buffer overnight with IgG and FOXO3 antibodies with agitation. Protein A agarose/Salmon Sperm DNA (Merck Millipore, Guyancourt, France) slurry was added and incubated for 2–4 h at 4 °C with agitation. The antibody-agarose complex was centrifuged and washed five times, and the immunoprecipitated fraction was eluted. The crosslinking was reversed by incubation at 65 °C for 4 h in the presence of 200 mM NaCl. The DNA was recovered by phenol/chloroform extraction and precipitated, and the abundance of specific sequence was measured by PCR using the corresponding primer sequences.

### The deletion of the promoter of *CTNNB1* by CRISPR/Cas9

The *CTNNB1* (Gene ID:1499) gene targeting sites in for sgRNA design was 5′-GCTTTCTCTATAAACATACTTGG-3′ and the primers were ordered from Sangon Biotech Company (Shanghai, China). The construction of the vectors and positive cells screening were performed as described previously^48^. Briefly, genomic DNA was phenol-chloroform extracted from G418-resistant HCC and CRC cell colonies. Cell identification was carried out in PCR reactions of 50 ng genomic DNA, 10 pmol of each primer (forward 5′-CACAGAGGTAACTTTCACTGCTG-3′ and reverse 5′-AAAGGCTGTGA ACTCTCCGTA-3′) and 0.5 unit of rTaq polymerase (Takara). PCR conditions were as follows; 5 min at 95 °C; 35 cycles of 30 sec. at 95 °C, 30 sec. at 62 °C, 40 sec. at 72 °C; and final extension at 72 °C for 5 min. All the positive clones were confirmed by Sanger sequencing.

### Generation of xenografts

NOD/SCID mice were obtained from the Animal Center of the Chinese Academy of Medical Science (Beijing, China). For generation of xenografts, 2 × 10^6^ HepG2, Huh7, HT29 and LoVo cells were injected subcutaneously into the NOD/SCID mice (n = 6). Five days later, the mice were grouped and administered intraperitoneally with DMSO or PS341 at a dose of 0.5 mg/kg two times per week for 30 days. The volume of xenografts was measured every five days (V = (π/6) (a × b × c)). Mice were sacrificed after 30 days.

In peritoneal spreading mice models, 2 × 10^6^ luciferase-expressing HepG2 and HT29 were intraperitoneally injected into the NOD/SCID mice (n = 5). Seven days later, the mice were grouped and administered intraperitoneally with DMSO or PS341 at a dose of 0.5 mg/kg two times per week for 35 days. Tumor growth was monitored by bioluminescence imaging using the Xenogen IVIS Lumina system (Xenogen Corporation, Hopkinton, MA) every week.

Animal work was permitted by the Institutional Animal Care and Use Committee of the Institute of Biophysics, Chinese Academy of Sciences and conducted in accordance with its recommendations and ethical regulations.

All experimental protocols were approved by the Animal Care and Use Committee, Institute of Biophysics, Chinese Academy of Sciences.

### Statistical analysis

Kaplan–Meier analysis was used to estimate the cumulative cause–specific survival rate and the differences in mouse survival with DMSO/PS341 (n = 8 ~ 11 per group). The influence of PS341 on the growth, apoptosis, migration, attachment, EMT, cell cycle, sphere formation and tumor formation of HCC and colorectal cancer cells was analyzed by the Student t test. In all statistical analyses, statistical significance in the two-sided test was indicated with *P* value of 0.05 or less and *P* value less than 0.01 was remarkably significant.

## Additional Information

**How to cite this article**: Yang, Z. *et al.* PS341 inhibits hepatocellular and colorectal cancer cells through the FOXO3/CTNNB1 signaling pathway. *Sci. Rep.* **6**, 22090; doi: 10.1038/srep22090 (2016).

## Supplementary Material

Supplementary Information
