# Supplementary material for: PS341 inhibits hepatocellular and colorectal cancer cells through the FOXO3/CTNNB1 signaling pathway
Source: Sci Rep. 2016 Feb 26;6:22090. doi: 10.1038/srep22090 (PMC4768146; doi:10.1038/srep22090)
Supplement: Supplementary Information [file srep22090-s1.pdf]

PS341 inhibits hepatocellular and colorectal cancer cells through the FOXO3/CTNNB1 signaling pathway

Zhao Yang[1, 2, 6*], Shengwu Liu[3, 4*], Mingao Zhu[5], Hong Zhang[5], Ji Wang[5], Qian Xu[5], Kaisu Lin[5], Xiumin Zhou[1], Min Tao[1], Chong Li[1, 6, †], and Hong Zhu[1, †]

[1]Department of Oncology, the First Affiliated Hospital of Soochow University, Suzhou, 215006, China.

[2]CAS Key Laboratory of Pathogenic Microbiology and Immunology, Institute of Microbiology, Chinese Academy of Sciences, Beijing 100101, China.

[3]Department of Medicine, Harvard Medical School, Boston, MA 02115, USA.

[4]Department of Medical Oncology, Dana-Farber Cancer Institute, Boston, MA 02215, USA.

[5]Department of Oncology, the Second Affiliated Hospital of Soochow University, Suzhou, 215000, China.

[6]CAS Key Laboratory of Infection and Immunity, Institute of Biophysics, Chinese Academy of Sciences, Beijing, China.

[*]These authors contributed equally to this work.

[†] Correspondence and requests for materials should be addressed to Dr. Chong Li, CAS Key Laboratory of Infection and Immunity, Institute of Biophysics, Chinese Academy of Sciences, Beijing 100101, China. Tel: +86-10-64884329, Fax: +86-10-64871293, Email: lichong@moon.ibp.ac.cn OR to Dr. Hong Zhu, Department of Oncology, the First Affiliated Hospital of Soochow University, Suzhou, 215006, China. Tel: +86-512-67780330, Fax: +86-512-67781716 215006, Email: zhuhong_jasmine@suda.edu.cn.

Key words: hepatocellular carcinoma (HCC), colorectal cancer (CRC), proteasome inhibitor PS341, tumor therapy

Running title: PS341 suppresses hepatocellular carcinoma and colorectal cancer cells

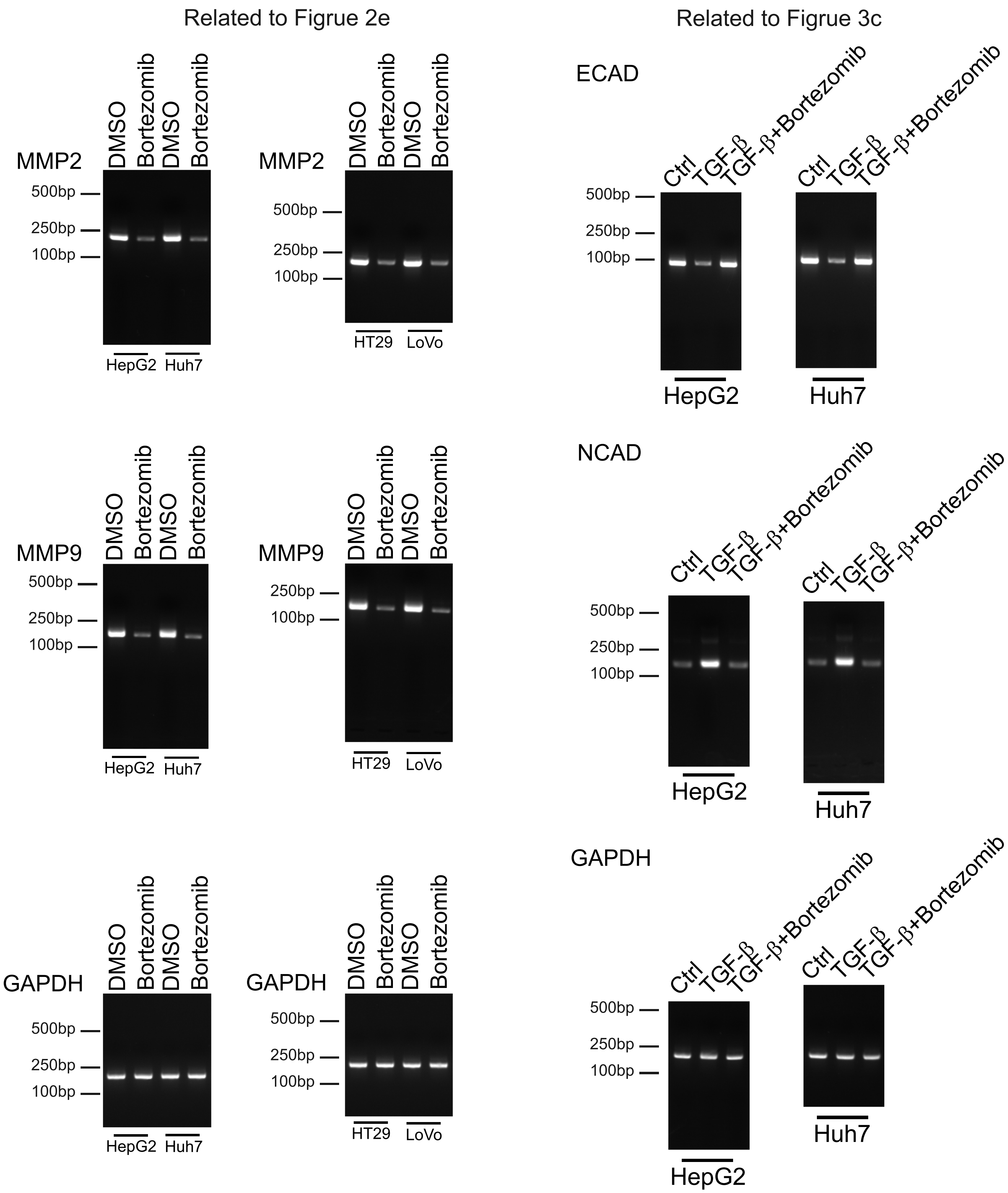

Supplementary Figure 1. Full-legth gels of Figure 2 and Figure 3.

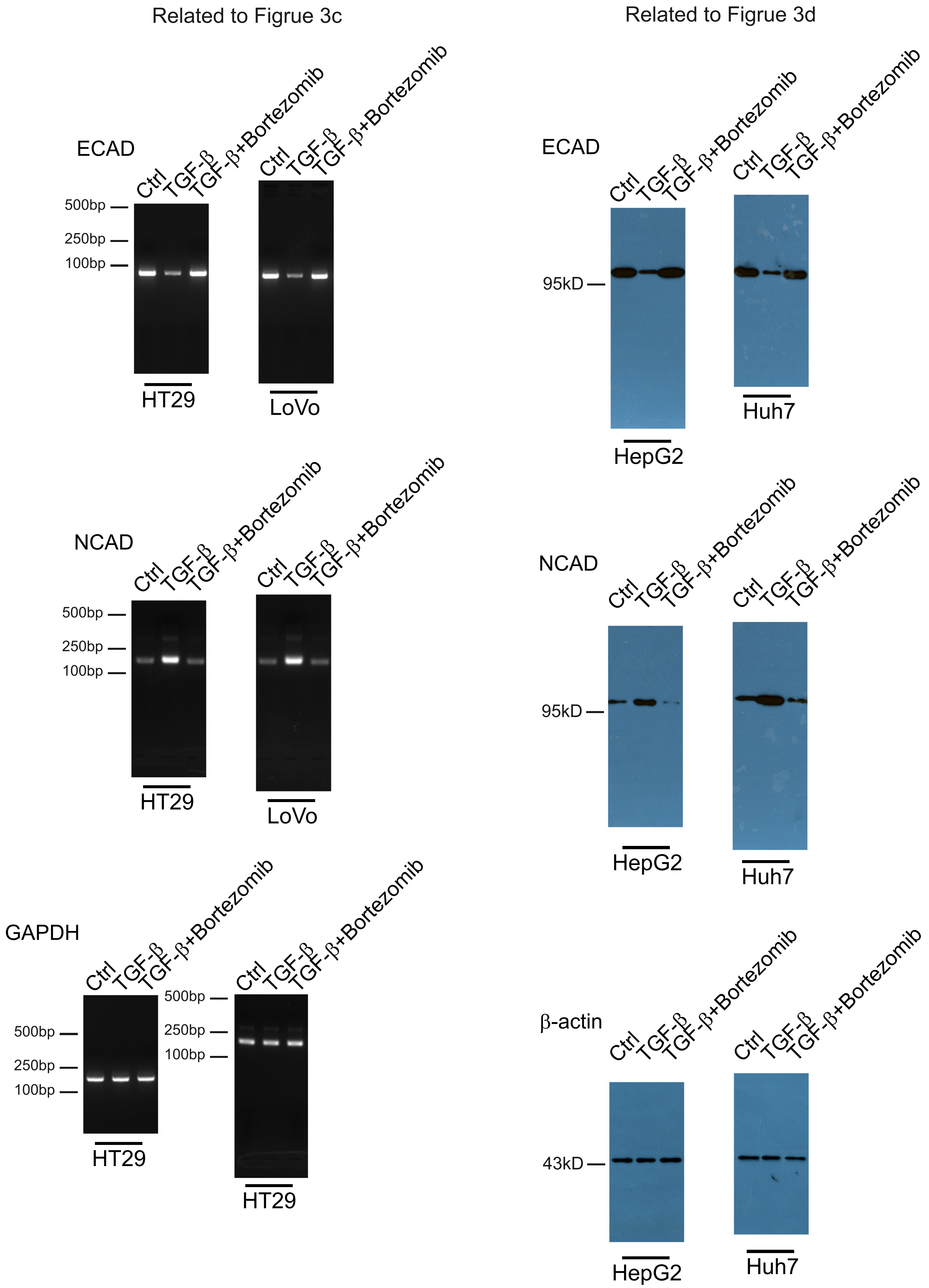

Supplementary Figure 2. Full-legth gels and blots of Figure 3.

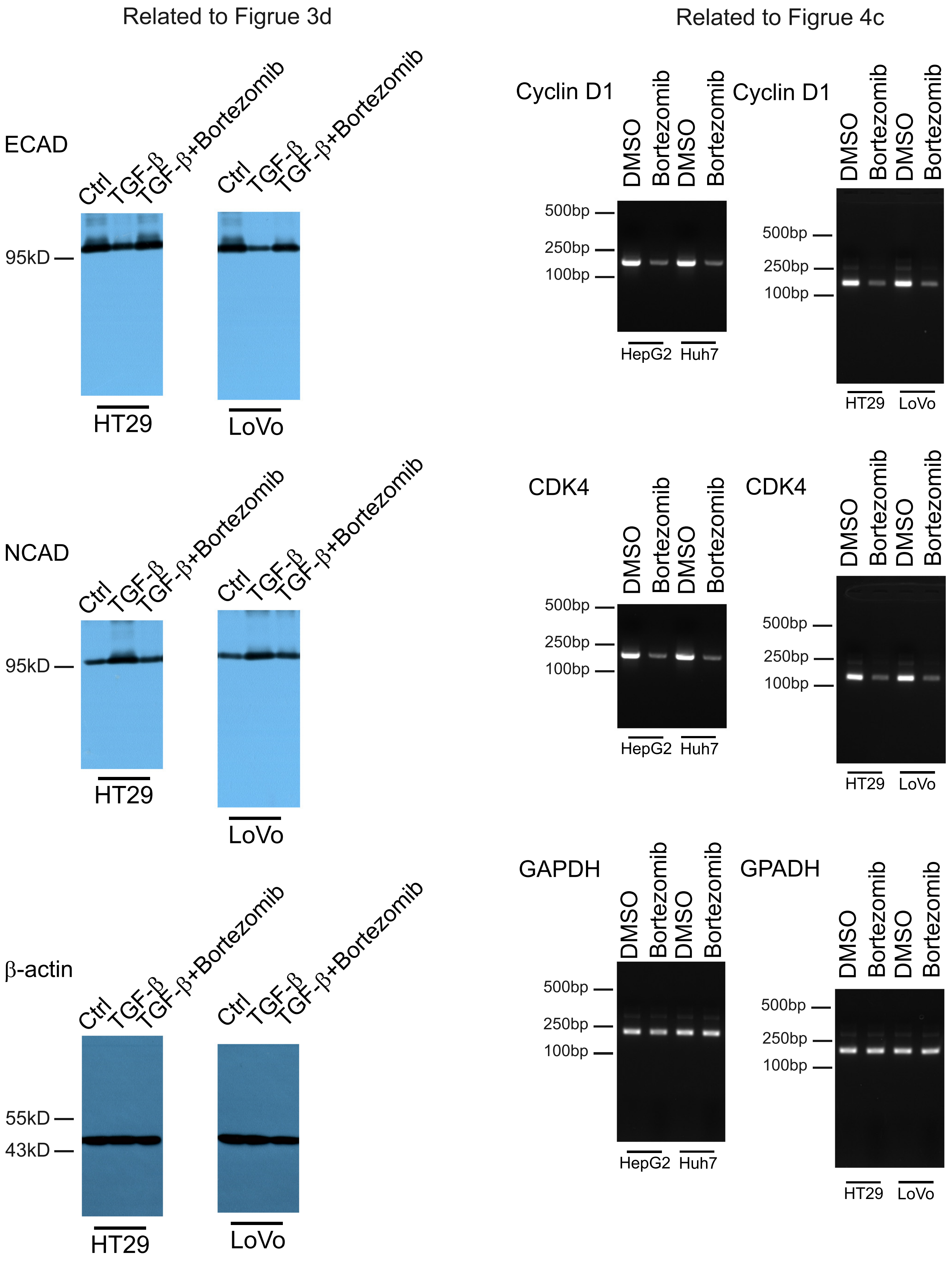

Supplementary Figure 3. Full-legth gels and blots of Figure 3 and Figure 4.

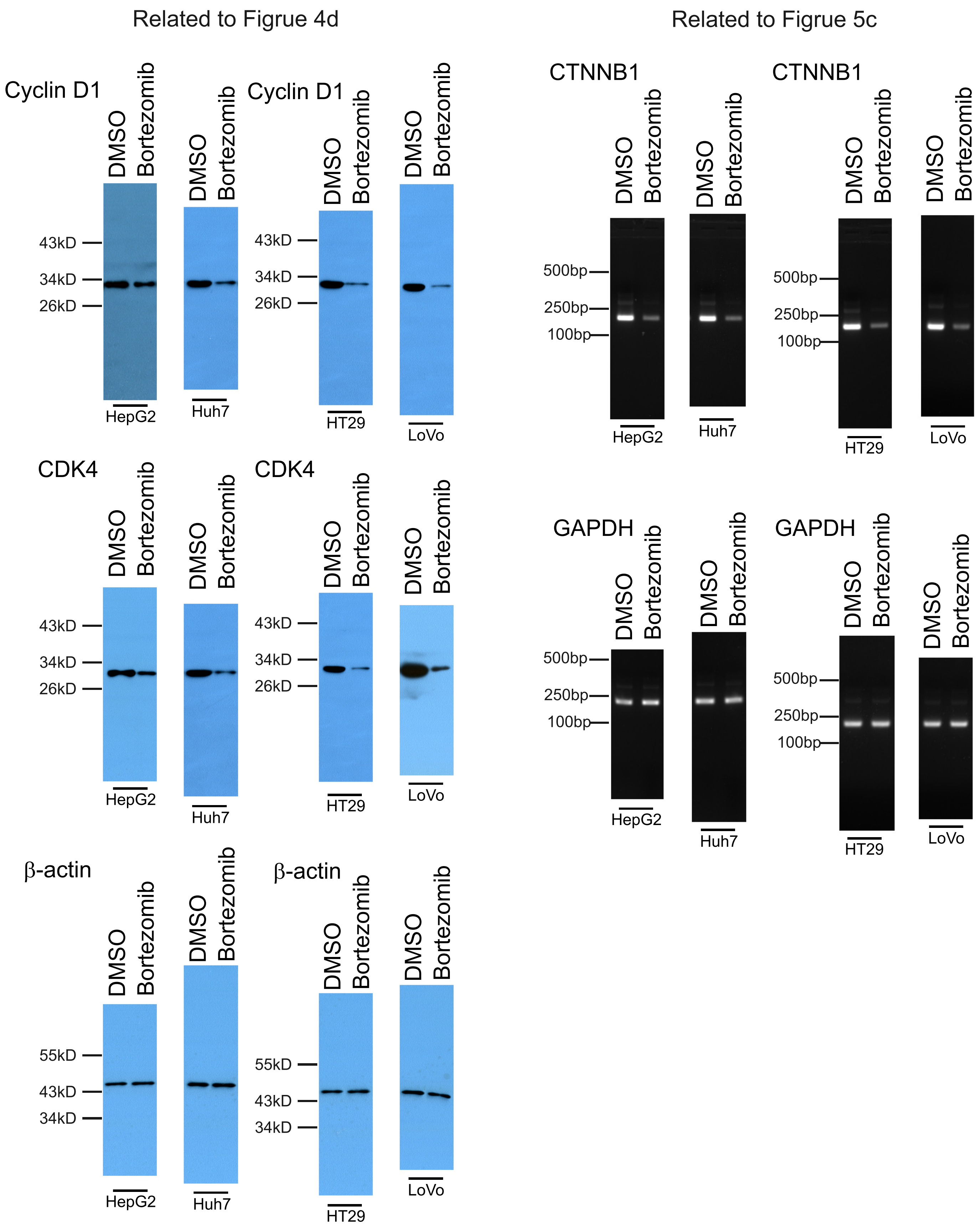

Supplementary Figure 4. Full-legth gels and blots of Figure 4 and Figure 5.

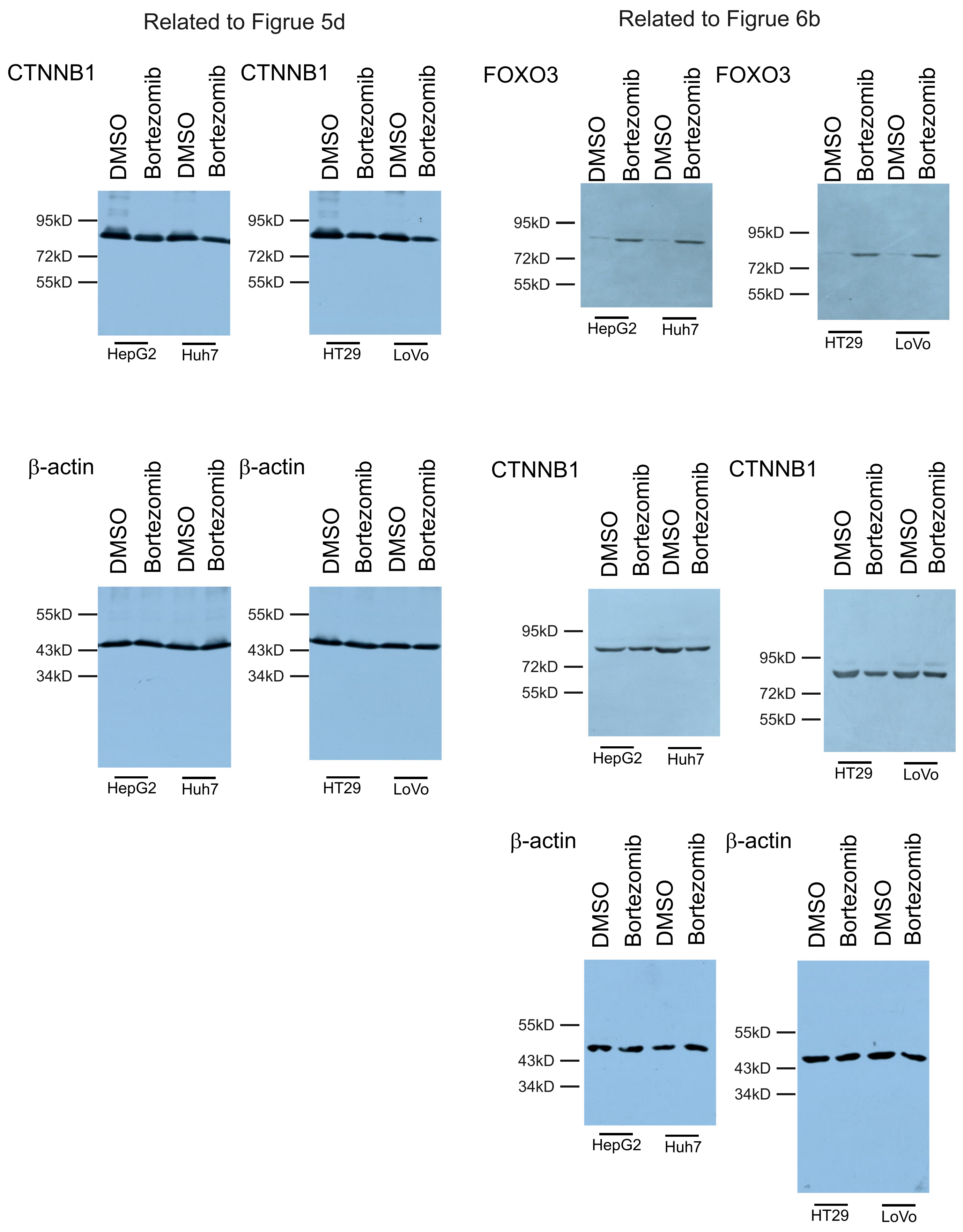

Supplementary Figure 5. Full-legth blots of Figure 5 and Figure 6.

Related to Figrue 6f

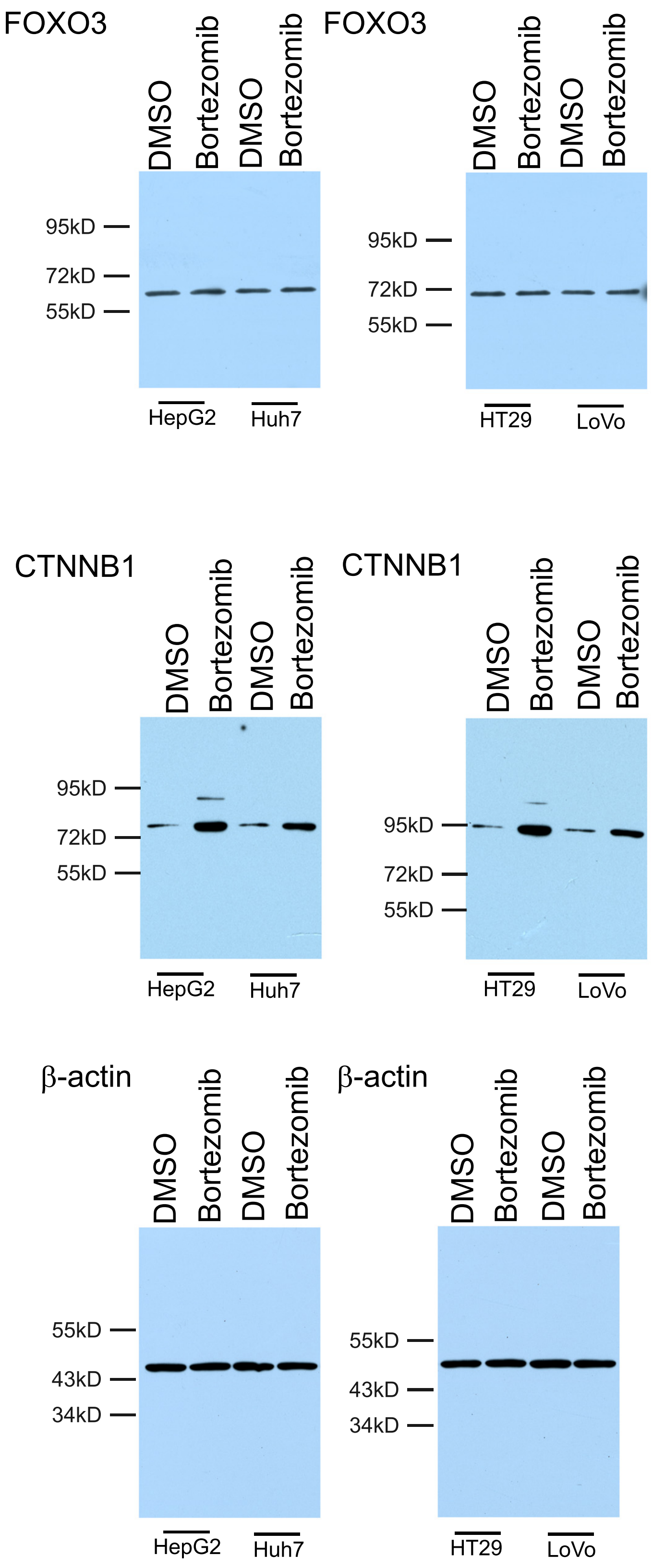

Supplementary Figure 6. Full-legth blots of Figure 6.

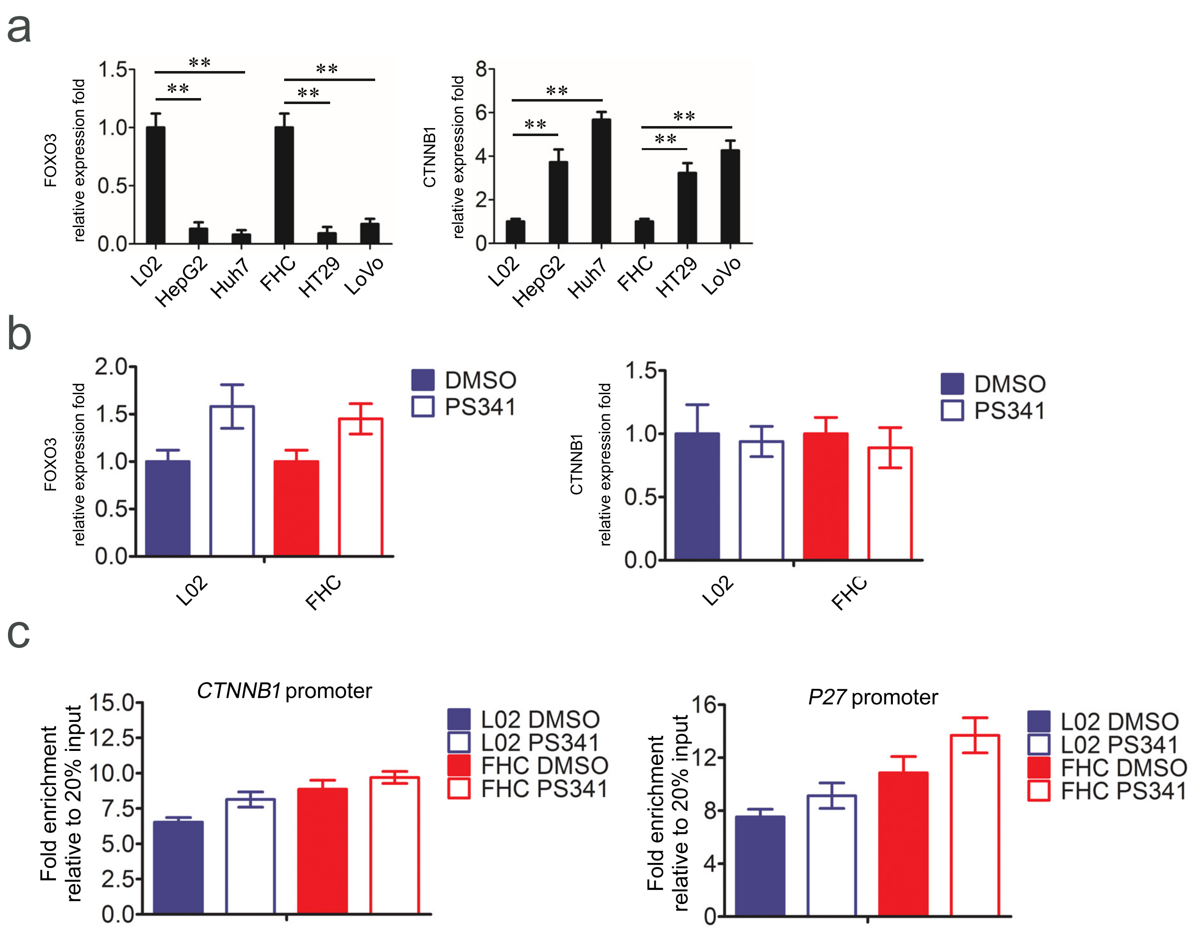

Supplementary Figure 7. (a) RT-PCR analysis of FOXO3 and CTNNB1 mRNA expression in L02, HepG2, Huh7, FHC, HT29 and LoVo cells. (b) The influence of PS341 treantment on FOXO3 and CTNNB1 mRNA expression levels in L02 and FHC cells. (c) Chromatin immunoprecipitation analysis of *CTNNB1* and *p27* promoter with IgG and FOXO3 antibodies in L02 and FHC cells with/without PS341 treatment. Data are displayed as mean ± SD. $**P<0.01$.

Supplementary Table 1. PCR primer pair sequences for selected genes.

Gene	Forward primer	Reverse primer	PCR	CHIP
MMP2	AGATCTTCTTCTTCAAGGACCGGTT	GGCTGGTCAGTGGCTTGGGGTA	○	×
MMP9	GCGGAGATTGGGAACCAGCTGTA	GACGCGCCTGTGTACACCCACA	○	×
GAPDH	AAGGTGAAGGTCGGAGTCAA	GGAAGATGGTGATGGGATTT	○	×
EACD	CCCACCACGTACAAGGGTC	CTGGGGTATTGGGGGCATC	○	×
NCAD	CAACTTGCCAGAAAACTCCAGG	ATGAAACCGGGCTATCTGCTC	○	×
VIM	TCCAGCAGCTTCCTGTAGGT	GAGAACTTTGCCGTTGAAGC	○	×
ZEB1	CAGCTTGATACCTGTGAATGGG	TATCTGTGGTCGTGTGGGACT	○	×
CD44	CTGCCGCTTTGCAGGTGTA	CATTGTGGGCAAGGTGCTATT	○	×
POU5F1	CTTGAATCCCGAATGGAAAGGG	GTGTATATCCCAGGGTGATCCTC	○	×
Cyclin A2	GGATGGTAGTTTTGAGTCACCAC	CACGAGGATAGCTCTCATACTGT	○	×
Cyclin B1	TTGGGGACATTGGTAACAAAGTC	ATAGGCTCAGGCGAAAGTTTTT	○	×
Cyclin D1	GCTGCGAAGTGGAAACCATC	CCTCCTTCTGCACACATTTGAA	○	×
CDK1	AAACTACAGGTCAAGTGGTAGCC	TCCTGCATAAGCACATCCTGA	○	×
CDK4	ATGGCTACCTCTCGATATGAGC	CATTGGGGACTCTCACACTCT	○	×
E2F1	ACGTGACGTGTCAGGACCT	GATCGGGCCTTGTTTGCTCTT	○	×
CTNNB1	AAAGCGGCTGTTAGTCACTGG	CGAGTCATTGCATACTGTCCAT	○	×
HES1	TCAACACGACACCGGATAAAC	GCCGCGAGCTATCTTTCTTCA	○	×
SMAD4	ACGAACGAGTTGTATCACCTGG	TGCACGATTACTTGGTGGATG	○	×
GLI1	AGAGTAGGGAATCTCATCCATCA	TGATGCAGTTCCTTTATTATCAGG	○	×
STAT3	ATCACGCCTTCTACAGACTGC	CATCCTGGAGATTCTCTACCACT	○	×
BMI1	CCACCTGATGTGTGTGCTTTG	TTCAGTAGTGGTCTGGTCTTGT	○	×
VGLL4	AACTGCAACCTCTCGCACTG	GCTCGGGCTCCTTGTAATTCT	○	×
FOXO3	ACCTTCGTCTCGAACTCCTTG	CTGTGGCTGAGTGAGTCTGAAG	○	×
SOX7	AGCCGGAGCAGACCTTCTT	GCCGGGGAGTAATAGGCAG	○	×
NOTCH1	CGCTGACGGAGTACAAGTG	GTAGGAGCCGACCTCGTTG	○	×

CTNNB1	TTTTCAGTCTATTGAAATGA	TAGATTCCATTTTTATCTGG	×	○
P27	GTCCCTTCCAGCTGTCACAT	GGAAACCAACCTTCCGTTCT	×	○
